# Identification and Functional Characterization of a Calcium-Sensing Receptor Mutation in an Infant with Familial Hypocalciuric Hypercalcemia

**DOI:** 10.4274/jcrpe.2800

**Published:** 2016-09-01

**Authors:** Anna Papadopoulou, Evangelia Gole, Katerina Melachroinou, Christos Meristoudis, Tania Siahanidou, Anastasios Papadimitriou

**Affiliations:** 1 Athens University Medical School, University General Hospital “Attikon”, Third Department of Pediatrics, Athens, Greece; 2 Biomedical Research Foundation of the Academy of Athens, Division of Basic Neurosciences, Athens, Greece; 3 University of Ioannina, Department of Biological Applications and Technology, Ioannina, Greece; 4 Athens University Medical School, “Aghia Sophia” Children’s Hospital, First Department of Pediatrics, Athens, Greece

**Keywords:** Familial hypocalciuric hypercalcemia, calcium-sensing receptor, calcium, hyperparathyroidism

## Abstract

Familial hypocalciuric hypercalcemia (FHH) is an autosomal dominant disorder, associated with inactivating mutations of the calcium-sensing receptor (CaSR). To evaluate the functional significance of a CaSR mutation, identified in a young infant who presented with hypercalcemia and hypocalciuria. The CaSR gene coding sequences were analyzed by polymerase chain reaction amplification and direct sequencing analysis. The mutation identified was introduced by site-directed mutagenesis into a wild-type (WT) CaSR plasmid, and human embryonic kidney 293 T cells were transfected with either the WT or mutant CaSR. The function of the mutated CaSR protein was analyzed by evaluating the free intracellular calcium [(Ca^2+^)_i_] response after challenge with extracellular calcium (Ca^2+^). We identified a heterozygous mutation c.772_773delGTinsA in exon 4 resulting in the substitution of amino acid valine (Val) with amino acid arginine (Arg) and the premature pause of the translation 46 amino acids later (Val258ArgfsTer47). Functional assay showed that cells transfected with the mutant CaSR had a significantly poorer response to extracellular Ca^2+^ stimulation compared with the WT. We have shown that the c.772_773delGTinsA mutation causes a significant alteration of CaSR function leading to features of FHH in an affected young infant since the first months of life.

**WHAT IS ALREADY KNOWN ON THIS TOPIC?**

Familial hypocalciuric hypercalcemia (FHH) is associated with inactivating mutations of calcium-sensing receptor (CaSR) gene. FHH, in most of the cases, is a benign condition featured by asymptomatic hypercalcemia, however, diagnosis is essential in order to avoid unnecessary parathyroidectomy.

**WHAT THIS STUDY ADDS?**

We describe the identification of c.772_773delGTinsA mutation of CaSR and the impact on the clinical phenotype in a young infant harboring the mutation. Our functional analysis shows that c.772_773delGTinsA mutation is associated with significant impairment of CaSR function leading subsequently to the phenotype of FHH.

## INTRODUCTION

The calcium-sensing receptor (CaSR) is a G protein-coupled cell surface receptor expressed abundantly in parathyroid chief cells (1) and renal tubular cells (2). CaSR holds a principal role in the maintenance of serum calcium (Ca^2+^) levels within a narrow range. Ca^2+^ is a mineral that plays a pivotal role in bone mineralization as well as in other physiological cell functions like intracellular signal transduction, hormone secretion, neurotransmitter release, and muscle cell contraction. The receptor recognizes any fluctuations in extracellular Ca^2+^ concentrations and finally modulates the parathyroid hormone (PTH) synthesis/secretion and Ca^2+^ renal reabsorption (3).

The human CaSR gene located in chromosome 3q13.3-21 contains 7 exons and encodes for a 1078 amino acid sequence (NM_000388). Exon 1 is untranslated, while exons 2-6 encode for a large extracellular N-terminal domain (ECD) and exon 7 for the seven transmembrane domains (TMDs) and the intracellular carboxy-terminal domain (ICD) (4). Loss-of-function mutations of the CaSR gene present in homozygotes with neonatal severe hyperparathyroidism (NSHPT), while heterozygotes develop familial hypocalciuric hypercalcemia (FHH) (5).

FHH is a rare autosomal dominant disorder, characterized by lifelong, usually asymptomatic hypercalcemia. The altered function of the receptor in this disorder decreases its sensitivity to extracellular Ca^2+^, shifting the set point of Ca^2+^-dependent PTH secretion to the right (6), therefore, FHH patients have higher PTH for their serum Ca^2+^ levels and they need to substantially increase their serum Ca^2+^ levels to suppress PTH secretion (7). Biochemical findings of patients with FHH include also very low urinary Ca^2+^ excretion and slightly elevated Mg_2_+ levels. Serum concentrations of vitamin D metabolites are usually normal irrespective of whether PTH levels are inappropriately normal or mildly elevated (8). Although in most of the cases the condition is benign, patients with FHH may develop complications like gallstones or acute pancreatitis (9).

In this study, we present the functional significance of a CaSR mutation identified in an infant with FHH who presented with asymptomatic hypercalcemia detected early in the infantile period.

## CASE REPORT

### Patient

A 4.5-month-old female infant of Greek origin was referred to our department in December of 2011 for evaluation of hypercalcemia detected in laboratory investigations performed during her hospital admission for urinary tract infection. She was born at term via normal vaginal delivery to healthy unrelated parents. She was growing well, her weight was at the 50^th^ percentile, and her psychomotor development was normal. The biochemical evaluation confirmed her hypercalcemia (Ca^2+^: 11.8 mg/dL; normal range: 9-11 mg/dL), accompanied by normal phosphorus (P: 5.9 mg/dL; normal range: 4-7 mg/dL), alkaline phosphatase (ALP: 360 U/L; normal range: 169-372 U/L), and albumin levels (Alb: 4.5 g/dL; normal range: 3.5-5 g/dL), while magnesium levels were slightly elevated (Mg_2_+: 2.5 mg/dL; normal range: 1.4-1.7 mg/dL). The 24 h urinary Ca^2+^ excretion was reduced with a significantly low Ca^2+^ to creatinine clearance ratio (CCCR) (urine Ca^2+^: 0.7 mg/kg/24 h; normal range: 2.8±0.66 and CCCR: 0.003; <0.01 suggests FHH, while >0.02 primary hyperparathyroidism). PTH levels were normal (PTH: 40 pg/mL; normal range: 10-80 pg/mL), while vitamin D levels were at the lowest range {25-hydroxyvitamin D [25(OH)D]: 21 ng/mL; normal range: 10-80 ng/mL}. Her mother’s laboratory evaluation revealed severe hypovitaminosis D [25(OH)D: 4 ng/mL] with normal Ca^2+^ (9.7 mg/dL; normal range: 8.9-10.1 mg/dL), P (2.8 mg/dL; normal range: 2.3-4.7 mg/dL), ALP (68 U/L; normal range: 20-130 U/L), and PTH levels (40.5 pg/mL; normal range: 10-80 pg/mL). It is interesting to note that the infant was exclusively breastfed and did not receive any vitamin D supplementation. Her father’s Ca^2+^ levels were at the upper normal range (Ca^2+^: 10.3 mg/dL), with normal P (2.66 mg/dL) and ALP (85 U/L). With the suspicion of FHH, mutational analysis of the CaSR gene was performed.

### Genetic Analysis

Genomic DNA was isolated from whole blood using the Purelink Genomic DNA kit (Invitrogen Ltd, UK). Exons 2-7 of CaSR gene (NM_000388) and their respective flanking regions were amplified, and polymerase chain reaction (PCR) products were sequenced on ABI 310 (Applied Biosystems, Foster City, CA, USA). Primers and PCR conditions are available upon request. Informed consent was obtained from the parents of the patient in order to pursuit the genetic analysis.

### Site-Directed Mutagenesis

The human CaSR cDNA cloned in the pCR 3.1 plasmid (pCR3.1/hCaSR) (Invitrogen) was kindly provided by Dr. Lia Baldini, University of Limoges, France. The GTinsA mutation was directly introduced to the wild-type (WT) CaSR plasmid to generate the mutated receptor using the QuickChange Site-Directed Mutagenesis kit (Stratagene, La Jolla, CA, USA) according to the manufacturer’s instructions. The set of primers overlapping the target regions of the WT cDNΑ were: 5’-CAGCATGTGGTAGAGAGATTCAAAATTCCAC-3’ and 5’- GTGGAATTTTGAATCTCTCTACCACATGCTG-3’.

### Cell Culture

Human embryonic kidney (HEK) 293 T cells were cultured in Dulbecco’s modified Eagle’s medium (Sigma; MO, USA) containing 10% fetal bovine serum (FBS), penicillin (100 U/mL), streptomycin (100 µg/mL), and 2 mM L-glutamine in a humidified incubator at 37 oC and a steady supply of 5% CO_2_. The cells were transiently transfected with CaSR cDNA (12 µg) in 10 cm tissue culture plates, using calcium phosphate DNA precipitates. Ca^2+^ measurements were performed 60 h post transfection.

### Free Intracellular Ca^2+^ [(Ca^2+^)_i_] Measurements

Sixty hours post transfection, cells were loaded with Fura 2-AM as already described (10). Cell pellets were suspended in Krebs-Ringer-HEPES (KRH) buffer [125 mM NaCI, 5 mM KCI, 1.2 mM KH_2_PO_4_, 1.2 mM MgSO_4_, 2 mM CaCI_2_, 6 mM D-glucose, and 25 mM HEPES-NaOH (pH 7.4)] containing 2% FBS counted in a hemocytometer and loaded with Fura 2-AM dye (5.0 µM), for 30 min at RT in the dark. Following loading, the cells were washed twice with CaCl_2_- deprived KRH and re-suspended in CaCl_2_-free KRH supplemented with 250 mM sulfinpyrazone to prevent dye leakage. Cell aliquots (1.5x10^6^) were transferred to a thermostatted cuvette (37 oC), maintained under continuous stirring, and analyzed in a Perkin-Elmer LS-55. The basal levels of (Ca^2+^)_i_ were measured upon addition of 1 mM EGTA and presented as nM. To investigate alterations in CaSR function, CaCl_2_ (3 mM) was reintroduced into the medium, and (Ca^2+^)_i_ increase was recorded.

### Statistical Analysis

All data are expressed as mean ± standard error of mean. Statistical analysis for multiple comparisons was performed using a one-way analysis of variance (ANOVA) followed by Tukey honest significant difference post-hoc test. Non-directional student’s t-tests were performed for comparisons involving only two groups. All statistical analyses were conducted using the Graph-Pad Prism software. Results were considered statistically significant at p≤0.05.

### Identification of the Calcium-Sensing Receptor Mutation

**Figure 1:**
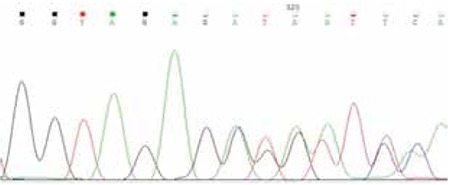
Sequencing analysis of CaSR gene (exon 4). Detection of c.772_773delGTinsA mutation in our proband

Direct sequencing of the coding region of CaSR gene revealed the heterozygous mutation c.772_773delGTinsA in exon 4 resulting in the substitution of amino acid valine (Val) with amino acid arginine (Arg) and the premature pause of the translation 46 amino acids later (p.Val258ArgfsTer47) (Figure 1). The same mutation was detected in the father but not in the mother. This change was not found in 50 unrelated individuals of the general population.

### Functional Analysis

**Figure 2:**
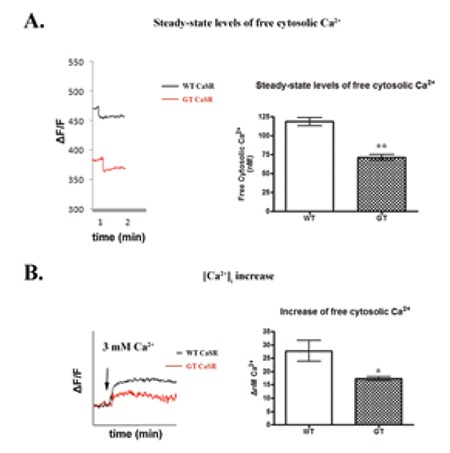
Alterations of Ca^2+^-evoked responses in the presence of CaSR mutation: (A) Steady-state levels of free cytosolic Ca^2+^. Representative Ca^2+^ traces are shown in the left panel and quantitative analysis of the (Ca^2+^)_i_ is depicted on the right panel. n=3; **p<0.01, comparing between wild-type and GT, using unpaired t-test. (B) Ca^2+^ influx upon stimulation with CaCl_2_. Representative Ca^2+^ measurements are shown in the left panel and quantitative estimation of (Ca^2+^)_i_ increase (ΔnM), upon CaCl_2_ stimulation is depicted on the right panel. n=3; *p<0.05, comparing between wild-type and GT using unpaired t-test. WT: wild-type

As already described, the WT and the mutant CaSR were transiently expressed in cultured HEK 293 T cells. The induction of (Ca^+2^)_i_ influx for both the WT and the mutant CaSR, was estimated after reintroduction of 3 mM CaCl_2_ (3 mM) into the medium. [Ca^+2^]_i_ increase was recorded as the net nM difference between the peak of (Ca^+2^)_i_ and the base-line (Ca^+2^)_i_ measurements. In Figure 2, it is shown that the WT over-expressing cells present a threshold of (Ca^2+^)_i_ of 118.6±5.590 nM. The cells harboring the c.772_773delGTinsA mutation exhibit significantly lower (Ca^2+^)_i_ (71.42±3.977 nM), p<0.01. Moreover, stimulation with exogenous addition of CaCl_2_ resulted in a significantly poorer response of the c.772_773delGTinsA over-expressing cells (17.33±0.7028 ΔnM) in comparison to WT (27.77±3.870 ΔnM), p<0.05.

## DISCUSSION

In this report, we studied the functional consequences of a CaSR mutation identified in an infant with mild hypercalcemia, admitted in our Pediatric Department. The genetic change was located in exon 4 of the CaSR gene and resulted in a new reading frame and theoretically, in a shorter CaSR protein. The same genetic change has been recently described in an adult patient with mild hypercalcemia and her two siblings originating from a Greek island (Nisyros) (11), however a functional assay was not performed to clarify the functional impact. No common ancestry is known between our patient and the patient from Nisyros.

The functional analysis showed that the mutant CaSR transfected into HEK 293 cells led to a statistically significant decrease of (Ca^2+^)_i_ influx after stimulation with exogenous Ca^2+^ compared with the WT. According to the known intracellular signaling pathway of CaSR, in response to binding of ligands to the receptor, the TMDs couple to G proteins and elicit several intracellular signaling cascades (12). These involve the activation of phospholipase C (PLC), breakdown of phosphatidylinositol 4,5-biphosphate, and formation of inositol 1,4,5-triphosphate (IP3) (13). Hence, high extracellular Ca^2+^ concentration stimulates the release of (Ca^2+^)_i_ from the intracellular stores which acts as a secondary messenger with different cell-dependent actions (14). Given that our mutation is located on the extracellular domain of the protein leading to a protein that lacks part of the ECD, the entire TMDs and the ICD possibly affect both ligand binding and cell signaling functions.

Approximately 130 inactivating mutations have been identified in patients with FHH, including mainly missense mutations. Most of them are clustered in the N-terminal domain and may alter the function of the receptor by affecting either the ligand binding or the G protein coupling (15). To our knowledge, 8 nonsense CaSR mutations leading to a shorter CaSR protein have been reported in the literature. These mutations have been described in exons 2 (1 mutation), 4 (2), 6 (1), and 7 (4) and are localized mainly in the ECD, while two mutations were found in the intracellular loop (ICL) [www.casrdb.mcgill.ca]. As these mutations usually lead to a severely truncated protein, one would expect to result in a more severe phenotype. However, in most of the reported cases, patients feature typical findings with mildly elevated Ca^2+^ (16,17) as was also observed in our case and her father as well as in the patients from Nisyros (11). Previous research has suggested that alterations caused by specific mutations depend on the location of the substituted amino acid in one of the critical regions for ligand binding, receptor trafficking, and signal transmission but not on the extend of the defect (13). Possible mechanisms by which inactivating mutations alter CaSR function include: incorrect folding and cellular trafficking of the protein thus resulting in reduced cell surface expression of the receptor (18); disruption of the disulfide bridge between ECL1 and ECL2 leading to abnormal secondary structure; abnormal conformational changes that follow ligand binding thus affecting G-protein coupling (19). It is also evident that missense mutations with a dominant negative effect, where the abnormal receptors dimerize with the wild types, result in a higher degree of hypercalcemia and a more severe phenotype than nonsense mutations producing considerably abnormal transcripts (16,17). This hypothesis can explain the mild phenotype observed in our patient although the identified CaSR mutation resulted in a truncated product of 303 aa instead of 1078. Unfortunately, we were not able to perform the Western blot analysis to further investigate the expression of CaSR.

Disorders of Ca^2+^ homeostasis featured by hypercalcemia are usually associated with hyperparathyroidism (HPT) either primary, secondary, or tertiary. The majority of cases of HPT are sporadic (95%), while only 5% are associated with a hereditary syndrome. Inheritable disorders include NSHPT, isolated familial HPT, multiple endocrine neoplasia syndromes (MEN-1, MEN-2A, MEN-4), HPT-jaw tumor syndrome, familial hypercalciuric hypercalcemia, and FHH (20). In the past years, patients with FHH misdiagnosed as primary HPT had inappropriately undergone parathyroidectomy without resolution of their symptoms (21). A recent consensus report of the European Society of Endocrine Surgeons clearly states that surgery is contraindicated in FHH, however, in some cases, the differential diagnosis from other conditions, where surgical management is necessary, can be challenging (22). In particular, the clinical picture of patients with FHH can be complicated by vitamin D deficiency which is widely prevalent. It is evident that vitamin D deficiency results in PTH elevation, and treatment is associated with normalization of PTH and lower Ca^2+^ levels (23). Our patient had insufficient vitamin D levels, while PTH levels did not exceed the normal range. Furthermore, although one of the typical features of FHH is hypocalciuria, some patients may present with hypercalciuria caused by the distinct functions of the mutated CaSR in renal and parathyroid cells leading to the incorrect diagnosis of primary HPT (24). Further, due to the tissue-specific CaSR-mediated signaling pathways, patients with FHH can manifest uncommon complications like pancreatitis, osteomalacia, and nephrolithiasis (24,25,26) that are not expected in this otherwise benign condition.

Hence, genetic evolution and the identification of mutations of the CaSR gene helped clinicians to distinguish FHH from other causes of HPT as reliable distinction is not always possible on clinical grounds. The aforementioned consensus report indicates that the diagnosis of hereditary HPT should be confirmed by genetic analysis and followed by genetic counseling of both patient and relatives. The diagnosis of FHH should be excluded in all patients before scheduling parathyroidectomy (22). However, as genetic analysis is expensive and not always easily available, strict selection criteria should be followed in order to avoid pitfalls and increase the sensitivity of CaSR mutational analysis (27).

Nevertheless, it is worth mentioning that CaSR mutations account for only 65% of FHH (28). Recently, germline inactivating mutations of GNA11, a gene encoding the α-subunit of a G protein coupled with CaSR during the pathway of signal transmission were found in patients with FHH type 2 (29). Moreover, mutations of AP2S1 were identified in patients with FHH type 3, a gene that encodes the adaptor protein 2 σ-subunit that is involved in CaSR endocytosis (30). Both these genetic defects result in impaired CaSR signaling leading to a similar clinical phenotype. Yet, as these mutations seem to be very rare (31), CaSR molecular analysis is considered to be an appropriate first step in the genetic evaluation of patients with features of FHH.

In the present study, we showed that the Val258ArgfsTer47 mutation in the CaSR gene identified in a young patient with hypercalcemia and her father resulted in a significant reduction of CaSR sensitization to extracellular Ca^2+^ leading to typical features of FHH. This is the second family of FHH reported from Greece and, interestingly, both families bear the same mutation. Molecular analysis of the CaSR gene, especially in patients who present early in life with hypocalciuric hypercalcemia, can establish the diagnosis of FHH and facilitate the subsequent clinical management.

## Ethics

Informed Consent: Informed consent was obtained from the parents of the patient in order to pursuit the genetic analysis.

Peer-review: Externally peer-reviewed.
